# Improving dementia diagnosis and management in primary care: a cohort study of the impact of a training and support program on physician competency, practice patterns, and community linkages

**DOI:** 10.1186/1471-2318-13-134

**Published:** 2013-12-10

**Authors:** Christine R Lathren, Philip D Sloane, Joseph D Hoyle, Sheryl Zimmerman, Daniel I Kaufer

**Affiliations:** 1 Program on Aging, Disability, and Long-Term Care, Cecil G. Sheps Center for Health Services Research, The University of North Carolina at Chapel Hill, CB# 7590, 725 Martin Luther King Jr. Blvd., Chapel Hill, NC 27599-7590, USA; 2 Department of Family Medicine, University of North Carolina, Chapel Hill, USA; 3 School of Social Work, University of North Carolina, Chapel Hill, USA; 4 Department of Neurology, University of North Carolina, Chapel Hill, USA

## Abstract

**Background:**

Primary care physicians routinely provide dementia care, but may lack the clinical skills and awareness of available resources to provide optimal care. We conducted a community-based pilot dementia training intervention designed to both improve clinical competency and increase utilization of local dementia care services.

**Methods:**

Physicians (N = 29) and affiliated staff (N = 24) participated in a one-day training program on dementia screening, diagnosis and management that included direct engagement with local support service providers. Questionnaires about their dementia care competency and referral patterns were completed before and 6 months after the training intervention.

**Results:**

Physicians reported significantly higher overall confidence in their dementia care competency 6 months post-training compared to pre-training. The largest reported improvements were in their ability to educate patients and caregivers about dementia and making appropriate referrals to community care services. Participants also reported markedly increased use of cognitive screening tools in providing care. Community service providers recorded approximately 160 physician-initiated referrals over a 2 year-period post-training, compared to few beforehand.

**Conclusions:**

Combining a targeted physician practice-based educational intervention with community service engagement improves dementia care competency in clinicians and promotes linkages between clinical and community dementia care providers.

## Background

More than one in eight adults over age 65 has dementia, and predictive models suggest a threefold rise in cases between 2000 and 2050 [1,2]. Primary care offices are responsible for medical management of most cases [3]. However, due to the often complex medical, social and behavioral needs of dementia patients, the average primary care physician has difficulty providing optimal dementia care [4,5]. Health care system barriers include insufficient time, insufficient support staff, difficulty accessing specialists, low reimbursement, and poor connections with community social service agencies [6]. Additionally, numerous studies have found that primary care physicians often lack knowledge or skill for appropriate screening, diagnosis and treatment of dementia [7,8]. These barriers often result in delayed or overlooked dementia diagnoses [4,7] and missed opportunities for treatment, care planning, and support for family members.

One of the most commonly overlooked areas associated with primary care management of dementia is lack of active partnerships between physicians and the various community resources available to families [9]. Many physicians report poor knowledge of resources available, unease with dementia counseling and family education, and overall low referral rates to social services [6,8-10]. Thus, community resources are often underutilized despite evidence that partnering primary care of dementia with these services can reduce nursing home admission rates [11], increase caregiver satisfaction with care [12], reduce caregiver depression [13] and improve the quality of care [14].

To address these challenges, a variety of physician education and quality improvement strategies have been developed. Examples include guideline implementation [12,15], care management support [12], academic detailing [16,17], and practice redesign [14]. These strategies have met with varying levels of success, with a major obstacle being how to engage physicians while addressing dementia in a sustainable and cost-effective manner.

This study examines the results of an interactive training and support program designed to educate primary care physicians and their staff on current evidence-based dementia clinical protocols and to link these practitioners to local dementia community resources.

## Methods

The project was conducted jointly by the Carolina Alzheimer’s Network (an educational outreach program of the University of North Carolina at Chapel Hill); North Carolina Area Agency on Aging (AAA) regions B (western NC, including Asheville), Q (eastern NC) and G (central NC, including Greensboro); and Project C.A.R.E. (Caregivers Alternative to Running on Empty; a support program for caregivers of persons with dementia operated by the State of North Carolina). This study was approved by the University of North Carolina at Chapel Hill Institutional Review Board, and all participants signed informed consent.

The three participating AAA regions totaled thirteen counties with a mix of urban and rural areas. As of 2010, these regions represented an estimated 200,000 people over age 65 [18].

Initial recruitment focused on physicians who were recommended by AAA staff and local geriatric physician leaders as being active in geriatric care. Additional names of active general primary care physicians were obtained via NC Medical Board licensee records, for a total of approximately 180 physicians invited to participate.

In regions B and Q, potential participants received a FedEx package containing a cover letter, brochure, and a faxed response form. In region G, recruitment letters were faxed only. Recruitment incentives included an honorarium and approximately 6 hours of continuing education credits. To foster team-based dementia care within practices settings, each physician was encouraged to bring a staff member to the training. Non-respondents were contacted by phone within 1 week, and project staff often spoke with office managers regarding participation. A total of 29 physicians were recruited; 7 from region B, 7 from region Q and 15 from region G.

Training sessions were conducted by two University of North Carolina medical school faculty members, a memory disorders specialist and a geriatrician, in conjunction with the local Project C.A.R.E. family consultant and AAA staff. Four Saturday trainings were provided, one each in regions B and Q (May 2009), and two in region G (March 2011).

The training program included sessions on evidence-based dementia screening, differential diagnosis, clinical assessment, pharmacological treatment, management of behavioral symptoms, caregiving, and community resources. These sessions were primarily discussion-based, and included cases provided by both the session leader and participants. Examples of cognitive tool administration and scoring were provided. All participants received a binder of materials containing clinical screening and assessment tools, relevant articles, and a laminated pocket card with information about dementia billing codes and Project C.A.R.E. contact information. These materials were also made available online [19]. As follow-up to the training, participants received e-newsletters every other month containing updates on dementia research and reminders about community resources.

During the community service session, participants were introduced to Project C.A.R.E. This resource provides dementia caregivers with a family consultant who offers in-home assessment, education, counseling, and funding up to $2,500 per year for caregiver respite services such as adult daycare and home aide visits [20]. Using case examples, participants learned how the family consultant can serve as a single point of entry for available dementia community services, depending on family needs and location. In addition to pocket cards with Project C.A.R.E. contact information, each office received a downloadable electronic link for making electronic referrals.

In order to foster continuing partnership and communication between primary care offices and community resource providers post training, Project C.A.R.E. personnel followed up with each participating office by phone and in-person. In regions B and Q, the Project C.A.R.E. consultant visited non-participating physicians’ offices to share information about Project C.A.R.E. services. Additionally, in both regions B and Q, a standardized feedback form was developed to provide physicians with the outcome of their referrals. In region G, an informal yet consistent process of e-mailed feedback was established between the family consultant and participating physicians.

Physician participants completed three questionnaires: a baseline interview approximately 1 month prior to the training, an evaluation immediately following conclusion of the training, and a follow-up questionnaire approximately 6 months post-training. In addition, a one year follow-up survey was administered to the 15 region G participants only. The baseline interview was conducted in-person by trained volunteer medical students or project staff. The physicians completed follow-up questionnaires online or in writing.

Pre-interview and follow-up questionnaires asked physicians to evaluate their confidence in areas of dementia diagnosis and management. The follow-up questionnaire asked physicians to describe their current dementia management behaviors such as use of cognitive assessment tools and frequency of referrals to community resources.

Data on number of physician referrals to Project C.A.R.E. were obtained from Project C.A.R.E. records.

## Results

Table 1 shows selected demographic and practice information about the physician participants. The typical participant was middle aged and had nearly 50% of their office visits from patients 65 and older. Estimates for number of patients with dementia or mild cognitive impairment were varied. Of the 29 physician attendees, 24 brought another office staff member such as a physician’s assistant, nurse practitioner, nurse, or medical assistant. Although office staff members were included in training, their survey results are not reported. Overall satisfaction ratings for the training were high, with an average score of 4.7 out of 5 (N = 19).

**Table 1:** Selected demographic and practice characteristics of physician participants (n = 29)

	**Percent or Mean (SD)**
% Male	59
Mean age (years) (n = 28)	52 (8.3)
Specialty (%)	
• Family Practice	65
• Internal Medicine	35
• Geriatrics Subspecialty or interest	21
Mean number of years in practice	20.8 (8.6)
Approximate percentage of patients in practice age 65 or older	46 (23.2)
Approximate number of patients physician sees in office with dementia (mean)	55 (72.8)
Approximate number of patients physician sees in office with mild cognitive impairment (mean)	73 (73.2)

### Physician competency and cognitive tools use

Physicians generally felt more confident in all areas of dementia clinical skills 6 months after training compared to before training (Table 2). Areas showing most improvement in self-rated confidence were distinguishing Alzheimer’s disease from other dementias, providing patient and caregiver education about dementia care, and referring patients to community resources. Responses showed good internal validity, with an alpha of 0.90.

**Table 2:** **Physician confidence in dementia care skills before and six months after training**^**a**^

**How confident are you in your ability to:**		**Pre-Training**^**b**^		**6 Month follow up**		**Change**	**p**
	**N**	**Mean**	**SD**	**Mean**	**SD**		
a. Screen patients for dementia?	28	3.64	1.03	3.93	0.81	+0.29	.12
b. Make a diagnosis of dementia?	27	3.59	0.97	3.85	0.86	+0.26	.11
c. Distinguish Alzheimer’s Disease from other forms of dementia?	28	2.50	1.04	3.21	0.96	+0.71	.001
d. Understand the value and use of assessment instruments for cognition?	28	3.29	1.08	3.93	0.90	+0.64	.006
e. Understand the role of MRI scans in the diagnosis of dementia?	28	2.75	0.97	3.39	1.13	+0.64	.015
f. Provide initial treatment to patients with memory loss?	28	3.43	0.92	4.07	0.72	+0.64	.003
g. Use medications for memory loss?	28	3.68	0.86	4.21	.69	+0.53	.003
h. Approach behavioral symptoms in patients with dementia?	28	3.14	0.80	3.61	0.74	+0.47	.010
i. Deliver patient and caregiver education about dementia care?	28	2.75	0.80	3.82	0.82	+1.07	<.001
j. Refer patients to community resources?	27	2.67	1.18	3.78	1.12	+1.11	.001
k. Disclose and explain a diagnosis of dementia to a patient?	28	3.43	0.88	3.86	0.80	+0.43	.005
l. Provide information to assist and respond to family caregivers of patients with dementia?	28	3.46	0.84	3.68	0.95	+0.22	.18

^a^Physicians were asked to rate their current confidence on a scale of 1 (not at all confident) to 5 (extremely confident).

^b^Change occurred from approximately 1 month prior to training compared to approximately 6 months following the training.

Participating physicians also reported increased use of dementia clinical screening and assessment tools post-training. On open-ended questions regarding cognitive assessment, the majority of physicians reported either only using the Mini Mental State Examination (31%, data not shown) (MMSE) [21] or a combination of MMSE and clock drawing (41%, data not shown) prior to the training, whereas post-training many physicians began using the Mini-Cog screening tool [22], the AD-8 caregiver assessment questionnaire [23], and an extended version of the MMSE designed to alert physicians to mild cognitive impairment [24]. The results from region G physicians who were specifically queried about their use of the Mini-Cog and AD-8 tools prior to and subsequent to the training are displayed in Table 3. Additionally, about half of the trained providers shared their new knowledge with other practice providers or staff.

**Table 3:** Physician practice patterns at 6 months post-training

**Physician use of cognitive assessment tools (N = 14)**^**a**^			
	Pre-training % currently use	6 month follow up % currently use	P^b^
Mini-Cog	21	93	.002
AD-8	7	57	.016
**Physician information sharing**^**c**^**(N = 28)**			
		% Reporting	
Other practice providers		50	
Other practice staff or nurses		54	
Providers in outside practices		21	

^a^Only 14 physicians were asked specifically about use of these tools at both baseline and follow-up.

^b^Exact test for related samples.

^c^Physicians were asked: In the months since the training, have you used the information gained to train, teach or consult with the following.

### Practice patterns and linkage to community resources

The 15 physicians in region G were asked additional practice pattern and community linkage questions at 1 year post-training (Table 4). In this group, 47% responded that the training influenced whom they screened for cognitive impairment. The most common changes in practice were screening for early signs of cognitive impairment and performing more screening evaluations based on patient age. The majority of physicians (87%) also reported changes in screening or assessment procedures, including changes in the tools they use and involving office staff in their administration. Nearly 50% of the trained physicians had designated a staff member to coordinate dementia care. Finally, nearly all participating physicians had made referrals to Project C.A.R.E. in the 6 month-period following training, with reported communication among the physician, family consultant, and referred family.

**Table 4:** **Physician practice patterns at 1 year post-training**^**a**^

**Changes in dementia screening and assessment practices**	**% Reporting**
Has who you screen for cognitive impairment changed since attending the dementia training? (N = 15)	47
Has how you administer cognitive screenings or assessments changed since attending the dementia training? (N = 15)	87
**Dementia Care Coordination**	
Is there a designated staff member in your office that helps coordinate care for people with dementia? (N = 15)	46
If yes, did that person attend the training? (N = 7)	100
**Physician Partnership and Communication with Project C.A.R.E.**	
In the past 6 months, have you made a referral to Project C.A.R.E.? (N = 15)	93
If yes, have you ever received feedback regarding a Project C.A.R.E. referral from the family consultant? (N = 14)	86
Have you ever received feedback regarding a Project C.A.R.E. referral from the family receiving services? (N = 14)	86

^a^Only the 15 Region G physicians received these questions at 1 year.

Examining the frequency of contact with community resource providers in the larger group of trained physicians, 20 of 27 responding physicians (74%) reported making a referral to community resources at least once a month in the 6 months post-training.

The combined efforts of training and outreach post-training resulted in approximately 60 new physician-initiated referrals in Regions B and Q and over 100 new physician-initiated referrals in region G over approximately 2 years. AAA staff from these regions state physicians had almost never provided aging service referrals prior to this project, and staff from region G did not recall ever having received a physician referral before the project began. These referrals resulted in various combinations of services coordinated through the Project C.A.R.E. family consultant, including caregiver education and counseling, and over 60 families receiving Project C.A.R.E. funding for respite services.

## Discussion

The results of this study suggest that a brief, intensive physician training program coupled with linkage to local community dementia service providers is a promising strategy for improving primary physician dementia care, particularly regarding use of effective screening and assessment tools and referral of families to community resources. These results were sustained over the course of the intervention, and were successfully implemented by non-academic community medical practices.

Among the most striking results of this project were its impact on promoting physician cognitive screening and assessment, and engendering previously non-existent physician-community resource partnerships. At 6 months post-training, there was a 72% increase in physicians’ use of the Mini-Cog, a screening tool with high sensitivity and specificity that has been shown to be cost-effective in primary care [25]. Moreover, it takes about 3 minutes and can be administered by trained non-physician staff. Similarly, use of the AD-8, a brief informant-reported questionnaire that complements direct measures (such as the Mini-Cog), also increased significantly. Among physicians surveyed one year post-training, most reported a higher rate of ongoing patient screening for possible dementia as a result of the training. Although not measured, the increased use of cognitive screening and assessment instruments would likely result in earlier detection of cognitive impairment, fewer missed diagnoses, and earlier referral to support services.

The level of communication between physicians’ offices and the Project C.A.R.E. family consultants remained relatively high well beyond the training for most participants. Efforts to provide consistent and systematic feedback to physicians regarding their referred patients appeared to encourage continued referrals, as has been noted by physician focus groups [9]. Furthermore, the “one-stop” community linkage approach successfully addressed some of the barriers medical providers face in partnering with community resources such as time constraints and lack of knowledge regarding available local services [6]. Having one active and familiar family consultant to direct families to a wide range of support services, from respite care to legal aid, allowed offices to spend less time in family counseling, simplified the referral processes, and “personalized” the partnership. The option to include office staff in the training sessions was important for improving efficiency, as office staff that received training were typically designated to make all dementia-related referrals.

While other studies have demonstrated increased referral rates from primary care providers, many of these interventions were more resource intensive. For example, Cherry et al. [12] found large increases in documented community resource referrals based on chart reviews, but the intervention involved a social worker serving as a case manager for participating practices. Reuben et al. [14] used prompts within a structured clinical visit note to remind physicians to make referrals, and required physicians and physician offices to agree to practice redesign and use of decision support tools. While the current intervention did employ some tools practices could integrate into their system of care, major restructuring was not required.

Other educational approaches to promote the use of available community resources have had varying results. An academic detailing intervention used a peer facilitator and community resource staff representative to deliver brief office-based dementia education to 104 primary care physicians and their office staff [16]. Similar to the present study, ratings for the effectiveness of the intervention in providing information on useful community resources were among the highest of all knowledge-type questions asked (over 70% of respondents chose “highly effective”). On follow up 3–6 months later, 55% of physicians acknowledged increased use of these resources, although actual use was not measured. However, only 35% of physician respondents indicated that their method of identifying dementia had changed. Thus, although this intervention reached more physicians, the overall impact on physicians’ screening and assessment practices was less robust.

Another recent primary care education interventional study that employed a 3-day dementia off-site “mini-residency” also reported significant gains in physician dementia clinical skills confidence and change in use of cognitive assessment tools [26]. Similar to the current intervention, training was led by peers in the field and included presentations by community resource providers. However, the time and cost-intensive nature of this intervention makes it a less feasible option for reaching a wider primary care physician audience.

There are several important limitations of this work. First, we relied on self-report and did not directly measure practice habits, except in the case of referral to Project C.A.R.E. Additionally, we did not measure the impact of training and community resource provision on families served. While research has shown that families linked to additional support services through their physician’s office can have improved outcomes [11-13], no specific data exists on connecting families to Project C.A.R.E. Linking this intervention to caregiver and patient outcomes will be an important focus of future work. Finally, although this study was conducted in geographically and socially diverse communities and practices, the sample size is modest. Recruitment of interested physicians was the most challenging aspect of this model. Low acceptance rates indicate that even with reimbursement for participating in training, this strategy may need to be altered to make it more appealing to a larger physician audience. Informal feedback from practices indicated that some primary care physicians, particularly those lacking significant geriatric populations, do not feel they come across dementia often enough in practice to warrant training. Additionally, authors speculate the time commitment of a weekend day long training was off-putting to some, and future trainings may need to be compacted. Higher response rates have been demonstrated for trainings that occurred on-site at the physicians’ offices, during breaks from patient responsibilities similar to drug representative education sessions [27]. However, the trade-off between training time and effectiveness is unknown.

It is also unclear to what extent the specific structure of the Project C.A.R.E. resource, including the availability of $2,500 per year respite funding, impacted referral rates. It is notable that in other North Carolina regions, Project C.A.R.E. respite dollars were stretched by offering $1,800 per family per year with minimal implications. Additionally, in region G, approximately 50% of the families referred to Project C.A.R.E. did not opt to use the respite funds, but did benefit from interactions with the family consultant. This suggests replication of this strategy in areas where the available community resources do not include flexible respite dollars might still be successful.

As previously reported in similar training programs [16,27], feedback from participants indicated high satisfaction, with an emphasis on a case-based and interactive training format, leadership by experienced physician peers, and inclusion of relevant handout materials. Furthermore, the partnerships established and ongoing communication between practices and community resources in this study are encouraging, as other resource intensive educational interventions in dementia care often do not outlast the study period. It is also notable that the majority of families who were referred to support services through their physician’s recommendation ultimately utilized these services. This supports the findings of Donath and colleagues [28] that a physician recommendation may be a strong motivator for caregivers to utilize available resources.

Finally, this paper adds to a body of growing research on strategies to improve dementia care through multi-faceted physician education interventions. Several recent educational approaches involve tailoring learning based on practice needs (“educational prescriptions”) [29] and using audit information to give specific and relevant feedback to trainees [17]. Other interventions involve mixed modes of educational delivery, including “blended learning” where online modules [30] or telephone coaching and decision support tools [31] supplement face-to-face trainings. In reality, the ability to scale up interventions to reach larger primary care audiences will likely depend on the flexibility of the intervention to meet varying physicians’ needs and time constraints. The availability of online learning options to supplement face-to-face training (or decrease the amount of time in face-to-face training) and tailored curricula to make participation more enticing and relevant will likely make dissemination more successful.

## Conclusions

This project demonstrates the potential impact of combining a targeted physician practice-based educational intervention with community service linkage in improving the dementia clinical care and access to support services. As primary care physicians face mounting pressure to provide quality and comprehensive care for an aging population, innovative and sustainable ways to train primary care physicians in dementia care are needed. Future work should include the expansion and dissemination of interventions to reach a larger primary care audience in a cost- and time-effective manner, while integrating protocols that measure the intervention’s direct impact on physician behavior and family outcomes.

## Competing interests

The authors declare that they have no competing interests.

## Author’s contributions

CL recruited participants, coordinated trainings, developed questionnaires, participated in the analysis and drafted the manuscript. PS secured study funding, designed the study, developed the training curriculum and trained the participants. JH recruited participants, participated in analysis and drafted the manuscript. SZ designed the study and developed the questionnaires. DK developed the training curriculum and trained participants. All authors read and approved the final manuscript.

## Pre-publication history

The pre-publication history for this paper can be accessed here:

http://www.biomedcentral.com/1471-2318/13/134/prepub
